# Dengue virus NS4B protein as a target for developing antivirals

**DOI:** 10.3389/fcimb.2022.959727

**Published:** 2022-08-09

**Authors:** Qingxin Li, Congbao Kang

**Affiliations:** ^1^ Guangdong Provincial Engineering Laboratory of Biomass High Value Utilization, Institute of Biological and Medical Engineering, Guangdong Academy of Sciences, Guangzhou, China; ^2^ Experimental Drug Development Centre, Agency for Science, Technology and Research, Singapore, Singapore

**Keywords:** dengue virus, NS4B, drug discovery, membrane protein, antivirals

## Abstract

Dengue virus is an important pathogen affecting global population while no specific treatment is available against this virus. Effort has been made to develop inhibitors through targeting viral nonstructural proteins such as NS3 and NS5 with enzymatic activities. No potent inhibitors entering clinical studies have been developed so far due to many challenges. The genome of dengue virus encodes four membrane-bound nonstructural proteins which do not possess any enzymatic activities. Studies have shown that the membrane protein-NS4B is a validated target for drug discovery and several NS4B inhibitors exhibited antiviral activities in various assays and entered preclinical studies.. Here, we summarize the recent studies on dengue NS4B protein. The structure and membrane topology of dengue NS4B derived from biochemical and biophysical studies are described. Function of NS4B through protein-protein interactions and some available NS4B inhibitors are summarized. Accumulated studies demonstrated that cell-based assays play important roles in developing NS4B inhibitors. Although the atomic structure of NS4B is not obtained, target-based drug discovery approach become feasible to develop NS4B inhibitors as recombinant NS4B protein is available.

## 1 Introduction

Dengue virus (DENV) belong to genus *Flavivirus* within the family of *Flavivirdae* that includes other important human pathogens such as Zika virus (ZIKV), yellow fever virus (YFV), Japanese encephalitis virus (JEV) and West Nile virus (WNV). DENV includes four serotypes (DENV-1-4) and is transmitted by mosquitos. DENV is estimated to cause over 390 million human infections annually and the number is still increasing (Bhatt et al., 2013). Dengue is an endemic disease in many countries affecting over 2.5 billion population living in tropical and subtropical regions where the conditions are suitable for the growth of mosquitos (Guzman and Harris, 2015; Obi et al., 2021). Dengue virus infection usually causes mild symptoms such as fever and does not need medical care. However, some infections can result in serious diseases such as dengue hemorrhagic fever and dengue shock syndrome which leads to approximately 22,000 deaths annually (Bhatt et al., 2013; Tuiskunen Bäck and Lundkvist, 2013; Obi et al., 2021). Vaccines have been developed to prevent dengue infection while the application is still limited because of many challenges (Lim et al., 2013; Villar et al., 2014). Therefore, it is necessary to develop antivirals to combat these viruses. Despite the efforts spent in drug discovery through targeting diverse targets (Rawlinson et al., 2006; Aleshin et al., 2007; Mass et al., 2007; Melino and Paci, 2007; Malet et al., 2008; Perera and Kuhn, 2008; Sampath and Padmanabhan, 2009; Noble and Shi, 2012; Brecher et al., 2013; Lim and Shi, 2013; Lim et al., 2013; Nitsche et al., 2014; Lim et al., 2015; Luo et al., 2015; Xie et al., 2015; Behnam et al., 2016), no drugs in clinics are available due to many challenges (Zou and Shi, 2019).

The viral genome is a single-strand, plus-sense RNA with approximately 11,000 nucleotides. The genome contains one open reading frame and encodes a polyprotein that can be further processed into 10 proteins by both host and viral proteases. These viral proteins include three structural proteins namely capsid protein (C), premembrane protein (prM) and envelope protein (E) and seven nonstructural proteins (NS1, NS2A, NS2B, NS3, NS4A, NS4B and NS5) (Pierson and Kielian, 2013; Li and Kang, 2022). The structural proteins are the key components of viral particles. C protein is critical for forming nucleocapsid. E and prM proteins are embedded in a lipid bilayer. The viral nonstructural proteins play indispensable roles in viral replication, virion assembly and evasion of host immune response *via* different mechanisms such as enzymatic activities or protein-protein interactions (Yon et al., 2005; Rawlinson et al., 2006; Lim et al., 2008; Nasar et al., 2020; Barnard et al., 2021). These nonstructural proteins participate in the formation of replication complex on the cell membrane where they can bind to RNA or proteins from both host and virus.

NS3 and NS5 are the well characterized viral proteins because they harbor enzymatic activities. NS3 contains both protease and helicase activities in its N-terminal and C-terminal domains, respectively (Prikhod'ko et al., 2002; Umareddy et al., 2006; Xu et al., 2006; Lescar et al., 2008; Luo et al., 2008b; Swarbrick et al., 2017). Its protease activity requires a cofactor region from NS2B. NS5 contains both RNA-dependent RNA polymerase (RdRp) and RNA methyltransferase (MTase) activities (Zou et al., 2011). Biochemical assays are available to characterize the activities of NS3 and NS5, which makes target-based drug discovery possible because the biochemical assays can be utilized in screening and evaluating the potency of inhibitors (Yin et al., 2006; Melino and Paci, 2007; Shiryaev et al., 2007a; Shiryaev et al., 2007b; Luo et al., 2008a). Quite a few inhibitors of NS3 and NS5 have been developed (Kroschewski et al., 2008; Zou et al., 2011; Kang et al., 2013; Poulsen et al., 2014; Lim et al., 2015; Dwivedi et al., 2020; Obi et al., 2021). It has been noted that there is no compound reaching into clinical stage due to lack of efficacy and unsatisfied chemical properties (Timiri et al., 2016; Nitsche, 2018; Lim, 2019). NS2A, NS2B, NS4A and NS4B are transmembrane proteins and the important components of the replication complex on the cell membrane (Li et al., 2015a; Li et al., 2015b; Li et al., 2016; Gopala Reddy et al., 2018; Lescar et al., 2018; Li et al., 2018). Among these proteins, NS2B plays a regulatory role in the protease activity of NS3 (Luo et al., 2008a; Robin et al., 2009; Noble et al., 2012; Noble et al., 2012) and others do not possess any enzymatic activities.

Although biochemical assays are not applicable to evaluate the activities of these membrane proteins, the inhibitors of these proteins have been identified by different strategies such as cell-based assays. An inhibitor of calmodulin was demonstrated to inhibit dengue virus through disrupting the molecular interactions between calmodulin and NS2A (Bautista-Carbajal et al., 2017). Inhibitors targeting NS2B were mainly derived from an artificial construct containing the regulatory region from NS2B and the N-terminal region of NS3. The available protease inhibitors bind to NS3 to prevent the substrate entering the active site or affect NS2B-NS3 interactions (Lescar et al., 2008; Steuer et al., 2011; Hammamy et al., 2013; Lim and Shi, 2013; Lim, 2019). Several NS4B inhibitors have been developed and are active in cell-based assays and animal models (Xie et al., 2015). In addition to cell-based assays, biophysical assays are also applied to explore the interactions between NS4B and inhibitors (Moquin et al., 2021). The success in developing NS4B inhibitors proves that it is a validated target (Wang et al., 2015; Zou B. et al., 2015). In this review, we summarize the structural studies on dengue NS4B, the roles of NS4B in protein-protein interactions, and the known NS4B inhibitors. Accumulated studies suggested the feasibility of developing antivirals through targeting NS4B.

## 2 Membrane topology and secondary structures of dengue NS4B

NS4B of flavivirus is a small membrane protein and contains over 200 amino acids with a molecular weight of approximately 27 kDa. NS4B is the largest nonstructural protein of flavivirus and contains many hydrophobic residues which are conversed among the four DENV serotypes. Dengue NS4B exhibits high similarity among four DENV serotypes and relatively low sequence homology to other viruses such as JEV, WNV, ZIKV and hepatitis C virus (HCV), which makes it challenging to develop broad-spectrum inhibitors through targeting NS4B. A study showed that dengue NS4B was N-glycosylated in virus-infection and glycosylation states of NS4B may play a role in viral replication (Naik et al., 2015). No crystal structure of NS4B is available so far, which may be due to the dynamic nature of this protein (Lescar et al., 2018). Its membrane topology was determined using a biochemical assay and secondary structural elements were determined using solution nuclear magnetic resonance (NMR) spectroscopy.

### 2.1 Membrane topology of NS4B

**Figure 1:**
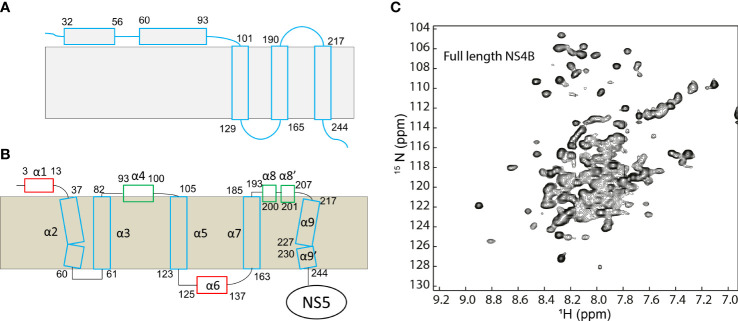
Topology of dengue NS4B protein. **(A)** Topology of NS4B determined using biochemical assays. **(B)** Topology of dengue NS4B in micelles determined using NMR spectroscopy. **(C)** The ^1^H-^15^N- heteronuclear single quantum coherence spectroscopy (HSQC) spectrum of dengue 3 NS4B in LMPG micelles. This figure is obtained from the reference (Li et al., 2016) with permission.

The membrane topology of dengue NS4B was determined using the following strategies (Miller et al., 2006). First, the transmembrane domains of NS4B were predicted by analyzing the amino acids with multiple programs. Second, the amino acids that interact with cell membrane were determined based on deletion mapping analysis. Lastly, protease protection assays were carried out to probe dengue NS4B topology. Dengue NS4B on the endoplasmic reticulum (ER) membrane contains two membrane association regions (pTMD1 and pTMD2) and three transmembrane regions (TMD3, TMD4 and TMD5) (**Figure 1**). The pTMD1 is formed by residues 32-56 and pTMD2 consists of residues 60-83. These two regions locate at the ER lumen side. TMD3 (residues 101-129), TMD4 (residues 190-165) and TMD5 (residues 217-244) are transmembrane helices to make the C-terminus facing the cytosol. The TMD5 may flip through the ER membrane after it is released from NS5 (Miller et al., 2006).

### 2.2 Secondary structure of NS4B

The structure of dengue NS4B in detergent micelles was explored using solution NMR spectroscopy (Li et al., 2015a; Li et al., 2016). Although the solution structure of NS4B was not determined due to lack of long-range distance restraints to define of the fold of the transmembrane helices in solution, the secondary structures of amino acids were determined based on the chemical shifts of backbone resonances. To study the structure of NS4B, both N-terminal domain and full-length NS4B were purified from bacterial cells and reconstituted in lyso-myristoyl phosphatidylglycerol (LMPG) micelles-a membrane system for folding of membrane proteins (Li et al., 2015a; Li et al., 2016). The result indicates that dengue NS4B in LMPG micelles contains eleven helical structures: α1 (E3-D13), α2 (S37-S60), α3 (T61-G82), α4 (P93-Y100), α5 (P105-G123), α6 (G125-R137), α7 (S163-M185), α8 (E193-G200), α8’ (P201-207), α9 (T217-R227), and α9’ (Y230-V244). Four helical elements including α1, α6, α8 and α8’ were not identified by sequence analysis in the previous study (Miller et al., 2006). Based on dynamics analysis and paramagnetic relaxation enhance experiments, α1 and α6 are solvent accessible while α8 and α8’ interact with the membrane (Li et al., 2015a; Li et al., 2016). Other helical elements are transmembrane helices (**Figure 1**). It has been noted that the topologies of DENV NS4B obtained from biochemical and structural studies are different as these experiments are carried out under different conditions. The biochemical studies identified the transmembrane regions in cells and the NMR study provided secondary structural information for these residues. Nonetheless, the secondary structures of NS4B will be helpful for understanding its function as well.

## 3 Function of NS4B through protein-protein interactions

The replication complex of DENV on ER membrane contains viral proteins and RNA. The roles of NS4B in the replication complex are achieved through protein-protein interactions (Wang et al., 2015; Xie et al., 2015; Tian et al., 2019; Fanunza et al., 2021; Lu et al., 2021). Researchers have identified several viral and host proteins that exhibited molecular interactions with NS4B. These interactions play critical roles in viral replication and response to host immune response.

### 3.1 Viral proteins

NS4B is located in the replication complex on the ER membrane where several viral proteins including NS1, NS2B and NS3 interact with NS4B (Long et al., 2020). The interactions are favoring to bring enzymes such as NS3/5 and viral RNA in close contact, which is critical for viral replication.

**Figure 2:**
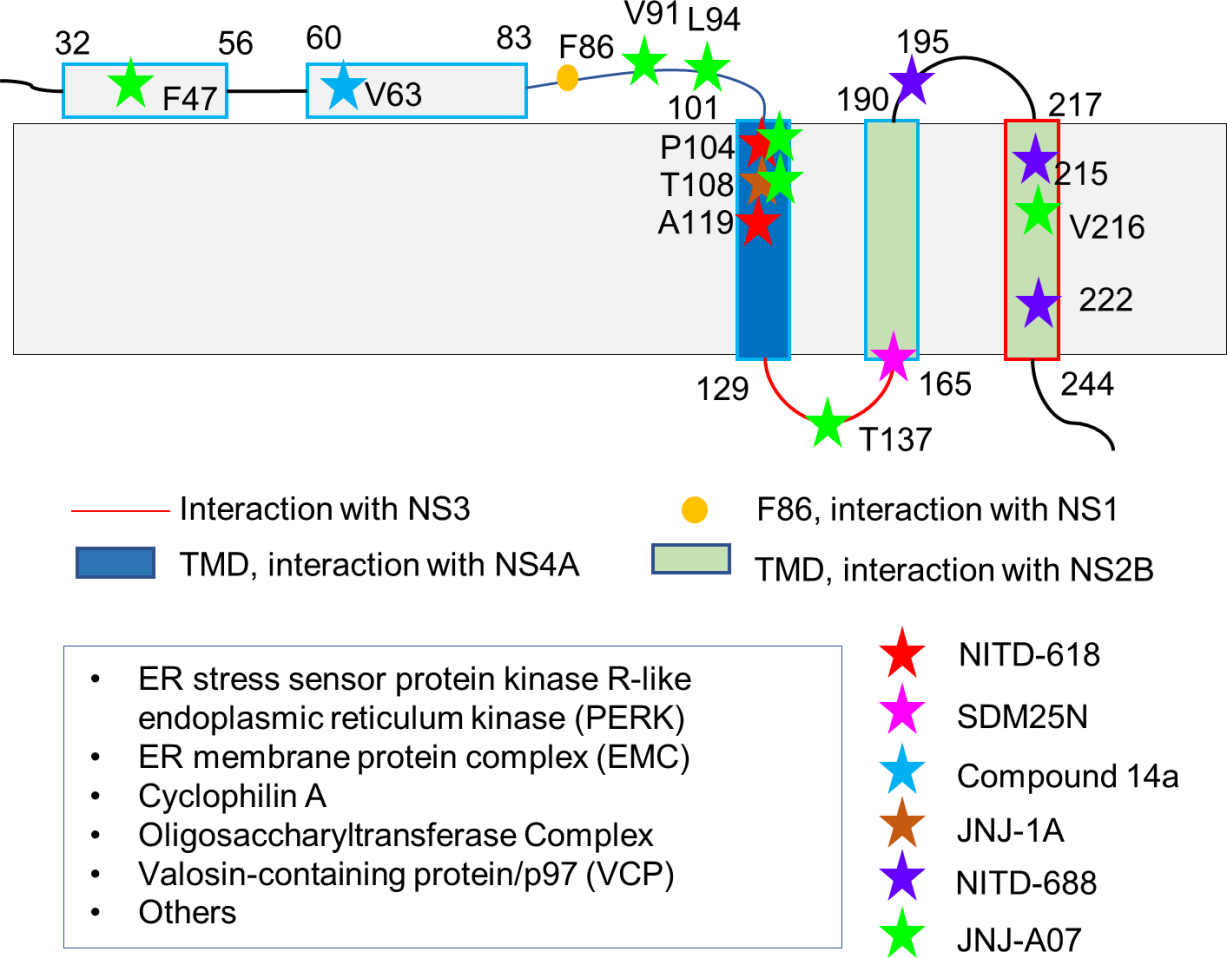
Residues of NS4B critical for inhibitor and protein bindings. The membrane topology of NS4B determined with biochemical assays is shown as this topology was used in most functional studies. Some residues involved in PPIs and inhibitor bindings are indicated and highlighted in different color. Some identified host proteins interacting with NS4B are listed in the box.

NS1 protein has multiple roles in viral replication such as counteracting antiviral immune responses, contributing to the severe clinical manifestations of dengue and affecting viral RNA replication (Muller and Young, 2013). NS4B may play a role in regulating the function of NS1 *via* direct protein-protein interactions. WNV NS1 was demonstrated to physically interact with NS4B based on the mass spectrometry and co-immunoprecipitation analysis. Genetic and biochemical assays suggested that F86 in NS4B is critical for interactions with NS1 (Youn et al., 2012). The result from several methods including bioinformatics, mass spectrometry analysis, and co-immunoprecipitation showed that dengue NS1 was modified with K48-linked polyubiquitin on several lysine residues during DENV infection (**Figure 2**). Ubiquitinated NS1 exhibited the reduced affinity to NS4B while abrogating ubiquitination of NS1 increased the NS1-NS4B interaction (Giraldo et al., 2018). NS1 forms homodimers and interact with cell membrane even though it does not contain any transmembrane regions. The interaction between NS1 and NS4 might be critical to stabilize NS1’s location on the membrane. NS1 and NS4B interactions may also include other viral proteins. A recent study took a combination of genetic, biochemical, and imaging approaches to demonstrate the interaction of NS1 with the NS4A-2K-4B, not matured NS4A or NS4B. The interaction is important for RNA replication (Płaszczyca et al., 2019).

Fluorescence resonance energy transfer (FRET) and bimolecular fluorescence complementation (BiFC) imaging assays demonstrated the close contact between WNV NS2B and NS4B (Yu et al., 2013). In the study, NS4B and other WNV membrane proteins were first demonstrated to localize on the ER membrane using immunofluorescence assay. Colocation of NS2B and NS4B was observed suggesting their direct interactions on the membrane. FRET assay indicated that NS2B and NS4B were within 10 nm and BiFC assay further proved their physical interaction. NS2B contains four transmembrane helices and a NS3-cofactor region that is forming a complex with NS3 protease domain. The transmembrane regions of NS2B may be involved in the interaction with NS4B. As NS2B is critical for the location of NS3 on the membrane, the NS2B-NS4B interaction might be critical for bringing the C-terminal region of NS3 in proximity with NS4B to form molecular interactions. Further studies on the regions that are important for NS2B-NS4B interaction will be useful for developing antivirals.

NS3 is a multifunctional protein with enzymatic activities. Its interaction with NS4B was first detected using a yeast two-hybrid assay and further proved with immunoprecipitation and pull-down experiments (Umareddy et al., 2006). NS4B was demonstrated to colocalize with NS3 and a double stranded RNA to enhance the dsRNA-unwinding activity of NS3 (Zou et al., 2015a). The residues from one of the cytoplasmic loops of NS4B critical for NS3 binding were identified using NMR spectroscopy (**Figure 2**). In addition, the binding affinity between NS4B and NS3 was determined by surface plasmon resonance (SPR) (Zou et al., 2015a). A recent study suggested that residues 51-83 of dengue NS4B accelerated unwinding duplex nucleic acids by NS3 using a FRET-based unwinding assay. A model of NS3-NS4B complex was built with the rigid-body docking, which predicted important regions for NS3-NS4B interaction. Further mutagenesis studies showed that changes in motif I (I190A) and motif III (P319L) of NS3 affected the unwinding activity stimulated by NS4B, suggesting that these residues are critical for the interaction (Lu et al., 2021). All the results suggest NS4B plays important roles in viral replication *via* binding to NS3 to affect its location and regulate its enzymatic activity.

To explore the function of the N-terminal region of NS4A, a construct in which the residues 27-34 of DENV-1 NS4A were replaced with those from JEV NS4A was made for analysis. The resulting virus was defective in replication while the recovered virus contained point mutation (T109I or L113F) in NS4B, suggesting that these residues might be critical for binding to NS4A (Tajima et al., 2011). Genetic interaction between NS4A and NS4B was further confirmed in JEV in which K79 mutation in NS4A was identified to be important for binding to NS4B. Mutations in NS4A (A97E) or NS4B (Y3N) were observed in recovered viruses, which further confirmed the residues involved in the interaction (Li X.D. et al., 2015). Physical interaction between dengue NS4A and NS4B was explored using biophysical studies, and their binding affinity was in nM range determined using recombinant proteins (Zou et al., 2015b). Residues critical for NS4A-NS4B interactions were also identified using NMR spectroscopy. Mutations in NS4A (L48A, T54A, and L60A) affecting its binding to NS4B abolished viral replication. These results demonstrated the relevance of NS4A-NS4B interaction to viral replication as their interactions might be critical for stabilizing the replication complex on the ER membrane (Zou et al., 2015a; Zou et al., 2015b).

Dengue NS4B forms homodimers, which was confirmed *in vitro* and in virus-infected cells. Due to the presence of transmembrane regions, the folding of purified recombinant dengue and WNV NS4Bs requires the presence of membrane systems such as dodecyl maltoside (DDM) detergent micelles. Cross-linking and light scatter experiments demonstrated that NS4B could form homodimers *in vitro*. Dimeric NS4B was also observed in the cells that were infected with DENV. The mutagenesis study demonstrated that two regions containing the cytosolic loop formed by residues 129 to 165 and the C-terminal region containing residues 166 to 248 are critical for NS4B dimerization (Zou et al., 2015a). The function of NS4B dimers is still unclear and it would be interesting to evaluate the impact of the known NS4B inhibitors on the dimerization.

### 3.2 Host protein-NS4B interactions

Studies have also demonstrated that Flavivirus NS4B proteins have molecular interactions with host proteins (**Figure 2**). These interactions play a role in evasion of host immune system or affect certain pathways in host cells. Studies have confirmed the molecular interactions between NS4B and several host proteins such as ER stress sensor protein kinase R-like endoplasmic reticulum kinase (PERK), ER membrane protein complex (EMC), cyclophilin A, the oligosaccharyltransferase (OST), and Valosin-containing protein/p97 (VCP).

PERK induces apoptosis under some conditions and a study showed that JEV NS4B plays important roles in the activation of PERK, which is related to JEV infection that triggers ER stress and neuron apoptosis (Wang et al., 2019). The co-immunoprecipitation experiment demonstrated the physical interaction of NS4B and PERK. One NS4B molecule interacts with two PERK molecules, indicating that NS4B is critical for PERK dimerization which is the active conformation to activate apoptosis. Two regions namely LIG-FHA (residues 162-168 and 192 -198) and the LIG-WD40 (residues 7-12, 31-36, 197-216, and 249-253) domains of JEV NS4B are critical to induce PERK dimerization and these regions are important for the interactions with PERK. In addition, the expression of JEV NS4B alone can induce encephalitis, which is dependent on the interactions with PERK in mice (Wang et al., 2019).

ER membrane protein complex (EMC) is critical for the insertion, stabilization or correctly folding of some transmembrane proteins (Chitwood et al., 2018; Guna et al., 2018; Shurtleff et al., 2018). CRISPR screen studies reveal the relationship between EMC and flavivirus proteins (Marceau et al., 2016). To further validate EMC enrichment identified in the flavivirus CRISPR screen (Zhang et al., 2016), isogenic EMC subunit knockout were generated in Huh7.5.1 and HEK293FT cell lines (Ngo et al., 2019). EMC was found to be important for the expression of dengue viral polyproteins and EMC may play a key role in the folding and stability of viral proteins. In the absence of EMC, degradation of NS4A-NS4B was observed. In addition, the physical interaction between EMC and NS4B was observed, suggesting that EMC is critical for the folding of NS4A-NS4B (Ngo et al., 2019). Another study showed that EMC played a role as a transmembrane chaperon for the folding of DENV and ZIKV membrane proteins such as NS4A and NS4B (Lin et al., 2019). The studies suggested that flaviviruses hijacked EMC for transmembrane protein biogenesis through NS4B. NS4B might be important to achieve optimal expression of their polyproteins through their interactions (Ngo et al., 2019).

To identify proteins that interact with NS4B, constructs containing glutathione S-transferases (GST) and cytoplasmic domains of YFV NS4B were purified from bacterial cells (Vidotto et al., 2017). GST pull-down assay was carried out against cellular extracts of HeLa cells. The samples were then subjected to separation with one-dimensional gel electrophoresis followed by liquid chromatography coupled to tandem mass spectrometry (Vidotto et al., 2017). Over one hundred human proteins were pulled down and cyclophilin A (CypA) was identified as a NS4B binder to regulate virus replication (Vidotto et al., 2017). Further study indicated that CypA inhibitors including sirolimus and cyclosporine A suppressed viral replication in the immunofluorescence and viral plaque assays. The results suggest that CypA plays a role in YFV replication through binding to NS4B and its inhibitors reduced viral replication which was dependent on this interaction (Vidotto et al., 2017). CypA contains peptidyl prolyl *cis-trans* isomerase activity and plays a role in protein folding and trafficking. Its interaction with NS4B may be critical for the folding of NS4B on the ER membrane.

The oligosaccharyltransferase (OST) complex was found to be essential for DENV infection in a CRISPR screen (Lin et al., 2017). Further experiments demonstrated that DENV did not require the canonical catalytic function of human OSTs, STT3A or STT3B while the OST subunit MAGT1 was necessary for DENV propagation. The study revealed that the CXXC motif in the oxidoreductase active site of MAGT1 was necessary for DENV propagation (Lin et al., 2017). Cells expressing an MAGT1 mutant in which CXXC is mutated into AXXA suppressed DENV infection. As conventional immunoprecipitation methods were not able to explore the interaction between STT3B/MAGT1 and NS4B, the proximity between MAGT1 and NS1 or NS4B was identified during DENV infection using engineered peroxidase APEX2 (Lin et al., 2017). Although further studies are needed to map NS4B and MAGT1 binding sites, the results exhibited the linkage between NS4B and OST in DENV infection. The oxidoreductase activity might play a role in managing correcting folding of NS4B or recruiting NS4B to a chaperone such as EMC. Further study to understand these interactions will provide a new strategy to develop antivirals (Lin et al., 2017).

Valosin-containing protein/p97 (VCP) is a hexameric ATPase playing regulatory roles in cellular functions such as ER-associated degradation (ERAD) and autophagy through extracting ubiquitylated proteins from their binding partners (Meyer et al., 2012; Tabata et al., 2021). VCP is an interesting target as its knockdown can result in the reduced production of DENV and JEV. A study showed that VCP ATPase activity was essential for the efficient replication of DENV. In the study, NS4B was shown to associate with and colocalize with VCP within DENV-induced convoluted membranes (Mazeaud et al., 2021). The function of VCP on JEV replication requires its interaction with its co-factors such as nuclear protein localization 4 (NPL4). A recent study showed the direct interaction between NPL4 and NS4B, which is necessary for the translocation of NS4B to the places of viral replication. The authors also demonstrated that a promoted stress granule formation by JEV and DENV was only observed in VCP inhibitor-treated cells. The expression of NS4B or VCP suppressed stress granule formation, suggesting that NS4B facilitates viral protein synthesis through recruiting VCP to the replication site to inhibit cellular stress responses (Arakawa et al., 2022).

Some other host proteins have also been identified to interact with NS4B. Using a yeast two-hybrid method, ubiquitin conjugating enzyme E2I (UBE2I) which can transfer SUMO protein to its protein targets, type II cytoskeletal 8 keratin (KRT8) and phosphoglycerate kinase (PGK1) were identified as NS4B binders (Khadka et al., 2011; Mairiang et al., 2013). Further studies are useful to confirm their interaction and map their binding regions.

### 3.3 Roles of NS4B in innate immunity

Flavivirus NS4B protein plays critical roles in evasion of host immune responses. Studies have shown that NS4B is an antagonist against host type-I interferon (IFN) signaling that is important for host defense against virus invasion through suppressing the phosphorylation of nuclear transport of STAT1/STAT2 (McNab et al., 2015; Cumberworth et al., 2017). Dengue NS4B was first demonstrated to block IFN signaling during viral infection (Muñoz-Jordán et al., 2003). The effect of NS4B on IFN through suppressing STAT1 phosphorylation upon ZIKV infection was also confirmed recently (Fanunza et al., 2021). The two membrane association regions (pTMD1 and pTMD2) are critical for the inhibitory function of NS4B on IFN signaling (Munoz-Jordan et al., 2005). NS4B proteins from DENV1/2/4 and WNV were shown to inhibit TBK1 phosphorylation and IFN-β induction (Dalrymple et al., 2015). Point mutations of residues in pTMD3 (position 116) of DENV NS4B exhibited various levels of IFN-α/β and IFN-stimulated gene expression, confirming the roles of NS4B in viral replication and viral adaption in different host cells such as human and mosquitos (Bui et al., 2018). Flavivirus NS4B was shown to inhibit the activity of stimulator of interferon genes (STING), which could be achieved *via* direct interactions (Ishikawa et al., 2009).

Accumulated studies have demonstrated that flavivirus NS4B plays important roles in viral replication through participating in the formation of viral replication complexes on the ER membrane (Miller et al., 2006; Lindenbach et al., 2007). NS4B is critical in the formation of stress granules (Arakawa et al., 2022), suppressing RNA interference *via* its TMD3 and TMD5 (Kakumani et al., 2013), modulating the unfolded protein response (Blázquez et al., 2015; Sepúlveda-Salinas and Ramos-Castañeda, 2017), and neutralizing innate immune responses such as type I IFN signaling, blocking the IFNα/β pathway (Green et al., 2014; Zmurko et al., 2015). All these functions prove that NS4B is a validated target for drug discovery against flaviviruses. Indeed, the availability of several potent NS4B inhibitors prove that NS4B is a validated target for developing antivirals against DENV and other viruses.

## 4 NS4B inhibitors

**Figure 3:**
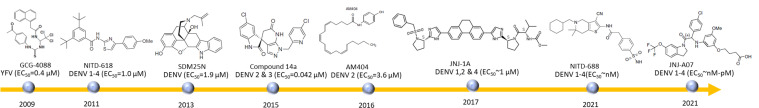
Some available NS4B inhibitors. During last several years, quite a few NS4B inhibitors have been designed. NITD-688 and JNJ-A07 are the most promising candidates for clinical studies due to their activities in mice and suitable PK parameters.

**Figure 4:**
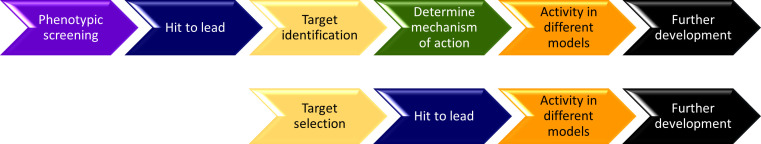
A schematic diagram of the steps during development of dengue NS4B inhibitors (upper panel). Lower panel shows the steps needed to develop NS4B inhibitors using a target-based drug discovery approach.

Although NS4B is a membrane protein which is challenging to set up *in vitro* assays to explore protein-inhibitor interactions, several NS4B inhibitors have been developed using phenotypic screening (Fikatas et al., 2021). The progress in developing NS4B inhibitors have been reviewed and highlighted (Xie et al., 2015; Ye et al., 2016; Lim, 2019; Behnam and Klein, 2021; Biering and Harris, 2021; Norshidah et al., 2021). A few NS4B inhibitors are described here (**Figure 3**; **Table 1**). The following steps were generally applied to identify NS4B inhibitors (**Figure 4**). A cell-based assay or phenotypic screening method was first applied to screen a compound library to identify a potent hit. Hit to lead was usually carried out based on the cell-based assay. Target deconvolution was then carried out to identify the lead binding target, which was usually achieved through inhibitor-induced mutation in the virus. Structure-activity relationship (SAR) of the inhibitors was usually determined using the cell-based assays. This is a time-consuming procedure while it is a feasible strategy in developing NS4B inhibitors.

**Table 1:** Some available dengue NS4B inhibitors.

Name	Screening method	EC_50_ (µM)	Mechanism of action	Current status	Reference
NITD-618	DENV-2 replication assay	1-4.1	Binding to NS4B (P104 and A119)	Research	Xie et al., 2011
SDM25N and AM404	DENV-2 replicon assay	1.9, 3.6	Binding to NS4B (F164)	Research	van Cleef et al., 2013
Compound 14a, JMX0254	DENV and HCV dual replication screening	0.042, 0.035-0.78	Binding to NS4B (V63)	Research	Wang et al., 2015; Xu et al., 2019
JNJ-1A	DENV-2 sub-genome assay	0.7	Binding to NS4B (T108)	Research	Hernandez-Morales et al., 2017
NITD-688	Phenotypic screening	0.008-0.038	Binding to NS4B (T195, T215 and A222).	Preclinical	Moquin et al., 2021
JNJ-A07	Phenotypic screening	nM to pM	Binding to NS4B to disrupt interaction with NS3	Preclinical	Kaptein et al., 2021

### 4.1 YFV NS4B inhibitors

The first reported NS4B inhibitors are active against YFV replication by targeting NS4B. A replicon-based assay with pseudoinfectious particles (PIPs) was developed to perform high-throughput screening (HTS) of a library consisting of 34, 000 compounds (Patkar et al., 2009). Any compounds exhibited a 50% effective concentration (EC_50_) less than 1 µM were selected for further studies. Among these identified hits, two compounds CCG-4088 and CCG-3394 with suitable selective index values exhibited an effect on viral replication and subjected to target deconvolution. Analyzing mutant viruses that escaped compound inhibition demonstrated that these two compounds may bind to NS4B at position 128 as K128R mutation was observed in the scape mutants (Patkar et al., 2009).

### 4.2 DENV NS4B inhibitors

#### 4.2.1 NITD-618

HTS of a library with 1.8 million compounds was carried out using a DENV-2 replicon assay system. NITD-618 was identified and exhibited a pan-DENV activity with EC_50_ values at a range of 1.0-4.1 µM (Xie et al., 2011). Interestingly NITD-618 was inactive against other RNA viruses such as WNV and YFV. This compound plays a role in suppressing RNA synthesis. Resistance studies revealed that NS4B was the target as mutations (P104L and A119T) were observed in the virus. Sequence analysis reveals that these two residues at positions 104 and 119 are conserved among these four dengue serotypes, but not in other flaviviruses such as WNV and FYV. The difference in these amino acids may account for the different activities observed. The residues critical for inhibitor bindings are also important for protein-protein interactions. Therefore, this compound might prevent NS4B from binding to other proteins while the poor pharmacokinetics makes it challenging for the further development (Xie et al., 2011; Xie et al., 2015).

#### 4.2.2 SDM25N and AM404

SDM25N-a δ opioid receptor antagonist was identified by screening a NIH Clinical Collection containing drug-like molecules using a DENV-2 replicon assay (van Cleef et al., 2013). This compound restricted RNA genome replication. Compound-resistant virus exhibited a mutation (F164L) in NS4B, indicating that the activity of SDM25N relies on NS4B. This compound only exhibited antiviral activity in human cells, not in mosquito cells, suggesting that it may interfere with NS4B’s activity on IFN (van Cleef et al., 2013). Another compound AM404 with an EC_50_ of 3.6 µM was identified using the similar method. Although no resistance mutations in viral genomes were observed, this compound may also bind to NS4B while further studies are required to confirm the interactions (van Cleef et al., 2016).

#### 4.2.3 Compound 14a

A spiropyrazolopyridone compound was identified with a similar strategy to NITD-618 (Wang et al., 2015). The compound was shown to be a DENV-2 and DENV-3 inhibitor with EC_50_s in a range of 10-80 nM (Wang et al., 2015). Further optimization resulted in compound 14a (Zou B. et al., 2015) and its resistance analysis revealed that residue 63 of NS4B could be the potential binding site. NS4B of DENV-2 and DENV-3 contains a valine residue at position 63 while NS4B of DENV-1 and DENV-4 has a leucine residue instead. This difference may explain the lack of activity of this compound against DENV-1 and DENV-2. It has been noted that the binding site of this class of compounds has been determined. The mechanism of action still needs to be confirmed by other experiments such as structural studies. It exhibited the activity in DENV-2-infected AG129 mice, indicating that it can be a potential preclinical candidate for further development (Wang et al., 2015).

#### 4.2.4 JNJ-1A

A DENV-2 sub-genome assay was applied to screen a compound library with anti-HCV activities (Hernandez-Morales et al., 2017). The identified compounds were further developed into JNJ-1A which exhibited the activity against DENV 1, 2 and 4. Resistance selection experiment using JNJ-1A identified the survival virus with a T108I mutation in NS4B, suggesting NS4B is the potential target. The absorption, distribution, metabolism, and excretion (ADME) analysis and the toxicity profile of JNJ-1A demonstrate its suboptimal drug-like properties. It is challenging to further enhance its activity due to a narrow SAR observed (Hernandez-Morales et al., 2017).

#### 4.2.5 NITD-688

Phenotypic screening of a Novartis compound library with 1.5 million compounds resulted in 13, 000 potent inhibitors identified (Moquin et al., 2021). Based on the EC_50_ values, the obtained compounds were further subjected to a selectivity assay. After a series of experiments, one tetrahydrobenzothiophene derivative was identified as a hit. Chemical modification improved the potency and solubility, which resulted in NITD-688 that exhibited EC_50_ values of 8-38 nM against all DENV serotypes. NITD-688 resistance virus exhibited mutations in NS4B. Mutations at residue 195 (T195A), residue 215 (T215S/A) and residue 222 (A222V) were observed, respectively (Moquin et al., 2021). Direct interaction of NS4B and NITD-688 was confirmed using NMR spectroscopy. This NS4B inhibitor is well characterized by biophysical method, confirming its direct interaction with NS4B. NITD-688 was shown to reduce viremia in AG129 mouse, indicating that it is potential to be applied in clinical studies (Moquin et al., 2021). It has been noted that this compound and NS4B interaction was well characterized through NMR studies, proving that NMR spectroscopy can play a role in developing NS4B inhibitors.

#### 4.2.6 JNJ-A07

A medium-throughput phenotypic screening of a library identified a compound which was further optimized into JNJ-A07 (Bardiot et al., 2018). JNJ-A07 is a potent pan-DENV inhibitor and exhibited nanomolar to picomolar activities against all the dengue serotypes including those clinical isolates (Kaptein et al., 2021). It took almost 40 weeks to generate resistant virus, suggesting the potency of the compound. Multiple mutations such as F47Y, V91A, L94F, P104S, T108I and T216N/P were observed in NS4B. Further pull-down study proved that JNJ-A07 blocked NS3-NS4B interaction. JNJ-A07 has a favorable pharmacokinetic profile based on data in both mice and rats (Kaptein et al., 2021). The activity of JNJ-A07 was also confirmed in mice (Kaptein et al., 2021). The potency and chemical properties of JNJ-A07 make it attractive for further studies (Biering and Harris, 2021).

## 5. Perspectives

### 5.1 Structural study of NS4B

Structure of a target protein is critical for rational drug design and understanding mechanisms of action of the inhibitors (Li and Kang, 2020a). The structure of NS4B is not available except that the secondary structure of NS4B determined using NMR spectroscopy (Li et al., 2015a; Li et al., 2016). Due to the dynamic and hydrophobic nature of NS4B, the following strategy can be applied in structural studies of NS4B. NMR is a very powerful tool to study structure of a membrane protein while it is time consuming (Kang and Li, 2011; Gayen et al., 2012). To study the structure of NS4B by NMR, a different membrane system such as bicelles and nanodisc will be useful as detergent micelles might not be an ideal membrane mimic to explore protein-protein/ligand interactions (Hilty et al., 2004; Fink et al., 2012). In addition, collection of long-range distance restraints is critical for determining the folding of NS4B, which can be achieved through other methods such as paramagnetic relaxation enhancement (Kang et al., 2008; Van Horn et al., 2009; Lu et al., 2012; Kroncke et al., 2016). Due to the molecular interactions between NS4B and other viral proteins, structural study of protein complex containing both NS4B and its binding partners will be possible using X-ray and cryo-EM while obtaining the protein complex with a high purity is challenging.

### 5.2 Determining residues of NS4B critical for protein bindings

Recombinant dengue NS4B has been obtained and NMR studies has been utilized to probe NS4B and inhibitor interactions (Li et al., 2015a; Zou et al., 2015b; Li et al., 2016). Recombinant dengue NS2B and NS4A are also available for structural studies (Huang et al., 2011; Lee et al., 2015; Li et al., 2015b; Li et al., 2018; Ng et al., 2019). Therefore, the residues of NS4B involved in protein-protein/ligand interactions can be determined using recombinant proteins. As membrane mimicking systems are needed for the folding of NS4B because of its hydrophobic nature, choosing a suitable membrane system for NMR or biophysical studies on NS4B is necessary as current NMR studies of NS4B adopted detergent micelle systems (Huang et al., 2011; Lee et al., 2015; Li et al., 2015b; Li et al., 2018; Ng et al., 2019). Other membrane mimics such as isotropic bicelles and nanodiscs will be useful for membrane protein-ligand binding studies.

### 5.3 Target-based discovery of NS4B inhibitors

All the NS4B inhibitors were identified through cell-based assays, which is time consuming because of the large effort spent in target deconvolution and understanding the SAR of the inhibitors (**Figure 4**). Target-based discovery of NS4B inhibitors is feasible because the recombinant dengue NS4B can be obtained and several biophysical methods to probe membrane protein-ligand interactions are available (Saenko et al., 1999). As mentioned previously, obtaining a membrane system suitable for biophysical studies is critical for a target-based drug design. In addition, fragment-based drug design (FBDD) can be applied in developing NS4B inhibitors (Lambruschini et al., 2013; Norton et al., 2016; Li and Kang, 2021). Different from HTS, FBDD can be utilized to develop NS4B inhibitors at a lower cost by starting from the hits binding weakly to NS4B (Li and Kang, 2017; Li and Kang, 2020b). Drug repurposing strategy can also be applied to identify NS4B inhibitors when reliable biophysical assays and screening strategies are available (Behnam and Klein, 2021).

### 5.4 Discovery of inhibitors targeting protein-protein interactions

NS4B binds to numerous proteins to play important roles in viral replication. Disrupting protein-protein interaction (PPI) was proposed to be a strategy for developing antivirals (Xie et al., 2015). The successful development of the inhibitor that affects NS4B and NS3 interaction proves the feasibility of this strategy. The same strategy can be applied to develop novel compounds disrupting the interaction between NS4B and other proteins, which can be achieved by setting up specific assays. It has been noted that it is challenging to develop such inhibitors disrupting protein-protein interaction due to many challenges such as lack of the druggable pocket. With the interactions involved in NS4B being characterized, suitable assays for identifying compounds disrupting such interaction can be developed.

### 5.5 Assays to identify NS4B inhibitors

Phenotypic screening and cell-based assays are very powerful for identifying inhibitors (Yokokawa, 2020). Some assays such as the virus replicon systems provide a reliable strategy to identify potent inhibitors. These assays can also be used to understand structure-activity relationship of the developed compounds. The development of potent NS4B inhibitors proves the importance of these methods. A lot of efforts have been spent to identify the target of an inhibitor derived from phenotypic screenings. Although generating mutants with drug resistance is a reliable method to identify the binding target, it is a time-consuming strategy. As several *in vitro* methods such as NMR spectroscopy are available to explore NS4B-ligand interactions, a combination of cell-based screening and *in vitro* binding assay will provide a reliable strategy to develop NS4B inhibitors. In addition to these methods utilized in hit identification, other strategies such as virtual screening can be applied (Moshiri et al., 2020) when the structure of NS4B is available.

## 6. Conclusions

Dengue NS4B is a validated target for antiviral development. The available inhibitors prove the feasibility of developing antivirals through targeting NS4B. In addition to cell-based assays to identify hits from a library, target-based drug discovery approach will be feasible for developing inhibitors with novel scaffolds because recombinant NS4B proteins and several biophysical assays are available. Determining the structure of dengue NS4B will be important for structure-based drug design, virtual screening, and understanding the mechanisms of actions of developed inhibitors. The availability of recombinant dengue NS4B proteins makes it possible to explore the structure.

## Author contributions

Conceptualization, QL and CK; writing—review and editing, QL and CK. All authors have read and agreed to the published version of the manuscript.

## Funding

QL appreciates the support from the “Hundred-Talent Program” (Grant Numbers: 2020GDASYL-20200102010 and 2020GDASYL-20200102009), Guangdong Academy of Sciences, GDAS’ Project of Science and Technology Development (2022GDASZH-2022010110), China.

## Acknowledgments

QL appreciates the support from Institute of Biological and Medical Engineering, Guangdong Academy of Sciences, Guangdong, China.

## Conflict of interest

The authors declare that the research was conducted in the absence of any commercial or financial relationships that could be construed as a potential conflict of interest.

## Publisher’s note

All claims expressed in this article are solely those of the authors and do not necessarily represent those of their affiliated organizations, or those of the publisher, the editors and the reviewers. Any product that may be evaluated in this article, or claim that may be made by its manufacturer, is not guaranteed or endorsed by the publisher.
